# Dose-dependent effects of NY-ESO-1 protein vaccine complexed with cholesteryl pullulan (CHP-NY-ESO-1) on immune responses and survival benefits of esophageal cancer patients

**DOI:** 10.1186/1479-5876-11-246

**Published:** 2013-10-05

**Authors:** Shinichi Kageyama, Hisashi Wada, Kei Muro, Yasumasa Niwa, Shugo Ueda, Hiroshi Miyata, Shuji Takiguchi, Sahoko H Sugino, Yoshihiro Miyahara, Hiroaki Ikeda, Naoko Imai, Eiichi Sato, Tomomi Yamada, Masaharu Osako, Mami Ohnishi, Naozumi Harada, Tadashi Hishida, Yuichiro Doki, Hiroshi Shiku

**Affiliations:** 1 Departments of Immuno-Gene Therapy and Cancer Vaccine, Mie University Graduate School of Medicine, 2-174, Edobashi, Tsu, Mie 514-8507, Japan; 2 Department of Gastroenterological Surgery, Osaka University Graduate School of Medicine, Yamadaoka 2-2 (E2), Suita, Osaka 565-0871, Japan; 3 Department of Clinical Oncology, Aichi Cancer Center Hospital, 1-1Kanokoden, Chikusa-ku Nagoya 464-8681, Japan; 4 Department of Endoscopy, Aichi Cancer Center Hospital, 1-1Kanokoden, Chikusa-ku Nagoya 464-8681, Japan; 5 Department of Gastroenterological Surgery and Oncology, Kitano Hospital, The Tazuke Kofukai Medical Research Institute, 2-4-20 Ohgimachi, Kita-ku, Osaka 530-8480, Japan; 6 Department of Anatomic Pathology, Tokyo Medical University, 6-1-1Shinjuku, Shinjuku-ku, Tokyo 160-8402, Japan; 7 Department of Translational Medical Science, Mie University Graduate School of Medicine, 2-174, Edobashi, Tsu, Mie 514-8507, Japan; 8 FiveRings, Co. Ltd, 9-4, 2-chome, Higashi-Tenman, Kita-ku, Osaka 530-0044, Japan; 9 ImmunoFrontier, Inc, 5-10, 2-chome, Sannou, Ota-ku, Tokyo 143-0023, Japan

**Keywords:** Esophageal cancer, Cancer vaccine, NY-ESO-1, Cholesteryl pullulan (CHP)

## Abstract

**Background:**

Cholesteryl pullulan (CHP) is a novel antigen delivery system for cancer vaccines. This study evaluated the safety, immune responses and clinical outcomes of patients who received the CHP-NY-ESO-1 complex vaccine, Drug code: IMF-001.

**Methods:**

Patients with advanced/metastatic esophageal cancer were enrolled and subcutaneously vaccinated with either 100 μg or 200 μg of NY-ESO-1 protein complexed with CHP. The primary endpoints were safety and humoral immune responses, and the secondary endpoint was clinical efficacy.

**Results:**

A total of 25 patients were enrolled. Thirteen and twelve patients were repeatedly vaccinated with 100 μg or 200 μg of CHP-NY-ESO-1 with a median of 8 or 9.5 doses, respectively. No serious adverse events related to the vaccine were observed. Three out of 13 patients in the 100-μg cohort and 7 out of 12 patients in the 200-μg cohort were positive for anti-NY-ESO-1 antibodies at baseline. In the 100-μg cohort, an antibody response was observed in 5 out of 10 pre-antibody-negatives patients, and the antibody levels were augmented in 2 pre-antibody-positive patients after vaccination. In the 200-μg cohort, all 5 pre-antibody-negative patients became seropositive, and the antibody level was amplified in all 7 pre-antibody-positive patients. No tumor shrinkage was observed. The patients who received 200 μg of CHP-NY-ESO-1 survived longer than patients receiving 100 μg of CHP-NY-ESO-1, even those who exhibited unresponsiveness to previous therapies or had higher tumor burdens.

**Conclusions:**

The safety and immunogenicity of CHP-NY-ESO-1 vaccine were confirmed. The 200 μg dose more efficiently induced immune responses and suggested better survival benefits. (Clinical trial registration number NCT01003808).

## Background

Complexes of cholesteryl pullulan (CHP) nano-particles that contain a tumor antigen are a new type of cancer vaccine with a novel antigen delivery system that presents multiple epitope peptides to both the MHC class I and class II pathways [1-4]. We have been developing CHP-protein human cancer vaccines that efficiently induce immune responses against multiple T cell epitopes for various HLA types. Previous clinical studies using CHP-HER2 and CHP-NY-ESO-1 vaccines showed that these vaccines could be administered repeatedly without serious adverse effects, and both vaccines induced antigen-specific CD4^+^ and CD8^+^ T cell immunity as well as humoral immunity [5-7].

Because the NY-ESO-1 antigen is a cancer-testis antigen that is exclusively expressed in the tumor tissue, aside from expression in the normal testis and placenta, this antigen is considered an ideal target for cancer immunotherapy [8,9].

The appropriate dose for NY-ESO-1 protein vaccine has not been determined, although doses up to 100 μg have been examined, in which a higher dose was more immunogenic compared to lower doses of 10 μg and 30 μg [10].

We conducted a dose-escalating trial with CHP-NY-ESO-1 vaccine doses of 100 μg and 200 μg for esophageal cancer patients who were resistant to standard therapies. We evaluated the safety and immune responses to the NY-ESO-1 antigen over the vaccination period, and explored the clinical impact on esophageal cancer patients with a poor prognosis.

In this study, we analyzed IgG antibody responses as antigen-specific immune responses. Although T cells that are induced by a cancer vaccine should be evaluated as an immune-monitoring marker, T cells can be difficult to detect directly and quantitatively assess, whereas IgG titers measured by ELISA could act as a suitable immune-monitoring marker. Analyzing antibody responses induced by CHP-NY-ESO-1 vaccine, the 200 μg-dose more efficiently induced immune responses and suggested better survival benefits.

## Materials and methods

### Preparation of CHP-NY-ESO-1 complex vaccine

CHP-NY-ESO-1 complex vaccine (Drug code: IMF-001) was provided by ImmunoFrontier, Inc. (Tokyo, Japan). All processes were performed following current Good Manufacturing Practices (cGMP) conditions. The toxicity of the drug products was assessed using animal models, and stability was monitored during the clinical trial using representative samples of the investigational drug product.

### Study design

This study was a phase 1, open-label, multi-institutional, dose-escalating clinical trial of the CHP-NY-ESO-1 complex vaccine administered subcutaneously to patients with unresectable, advanced, or refractory esophageal tumors that expressed the NY-ESO-1 antigen. The primary objective was to determine the maximum tolerated dose (MTD) and the biological recommended dose, and the secondary objective was to assess clinical efficacy.

Patients were eligible for entry, if they had a performance status of 0, 1, or 2, were at least 20 years old, had a life expectancy of 4 months or more, and did not have impaired organ function. Patients were ineligible if they were positive for HIV antibody, had multiple cancers, autoimmune disease, serious allergy history, or active brain metastasis, or received previous chemotherapy, systemic steroid or immunosuppressive therapy within less than 4 weeks.

The patients were divided into the following two cohorts of 10 patients each: Cohort 1, 100 μg of the NY-ESO-1 protein every two weeks, and Cohort 2, 200 μg of the NY-ESO-1 protein every two weeks. When a patient withdrew from the trial within three vaccinations, they were replaced with an additional patient.

Clinical responses were assessed according to the Response Evaluation Criteria in Solid Tumors (RECIST ver1.1) [11] and its modified version. The modified version is based on immune-related Response Criteria (ir-RC) [12] and includes the following: Tumor responses were assessed every 6 weeks. Even if disease progression was observed within the first 12 weeks, PD (progressive disease) was not judged. When disease progression was observed after 18 weeks, PD was determined.

Each patient received 6 administrations. However, the treatment could be continued beyond this period if the patient wished to maintain treatment and met the following criteria: 1) no evidence of tumor progression or worsening of performance status (PS), and 2) an anti-NY-ESO-1 antibody response was confirmed. Safety was evaluated according to the National Cancer Institute Common Terminology Criteria for Adverse Events ver.3.0 (NCI-CTCAE ver.3.0) [13]. All the safety information was collected and evaluated, and dose escalation was judged by the Independent Data and Safety Committee.

The study was performed in accordance with the current version of the Declaration of Helsinki and Good Clinical Practice. Written informed consent was obtained from all patients participating in this study. The protocol was approved by the institutional review board at each site. The clinical trial was sponsored by ImmunoFrontier, Inc. (Tokyo, Japan), and registered as ID: NCT01003808 of ClinicalTrials.Gov.

### Expression of NY-ESO-1 antigen

NY-ESO-1 expression was assessed by immunohistochemistry with the monoclonal antibody, E978 (Sigma-Aldrich, Saint Louis, MO), [9] or quantitative RealTime-PCR (qRT-PCR) using specific primers [14].

### Serum samples

To analyze antigen-specific antibody responses, sera were collected at baseline and two weeks after each vaccination. All sera were stored at −80°C until analysis.

### Antibody responses to NY-ESO-1 antigen

NY-ESO-1-specific antibodies in the sera were measured by ELISA as described previously [15]. Briefly, recombinant NY-ESO-1 protein*s* (His-tag and GST-tag) and NY-ESO-1 peptides were absorbed onto immunoplates (442404; Nunc, Roskilde, Denmark) at a concentration of 10 ng/50 μL/well at 4°C. The collected serum samples were diluted from 1:400 to 1:102,400. After washing and blocking the plate, the sera were added and incubated for 10 h. After washing, goat anti-human IgG (H + L chain) (MBL, Nagoya, Japan) conjugated with peroxidase (The Binding Site, San Diego, CA) was added. After adding the TMB substrate (Pierce, Rockford, IL), the plate was read using a Microplate Reader (model 550; Bio-Rad, Hercules, CA).

Serum samples for 80 healthy volunteers were evaluated to determine a cut-off level for the anti-NY-ESO-1 antibody based on the optical density (OD)_450–550_ absorption value. The cut-off level of anti-NY-ESO-1 IgG was 0.182. A sample was considered to be positive for anti-NY-ESO-1 antibodies if the optical density (OD)_450–550_ absorption value in the ELISA was at the cut-off level or higher at a serum dilution of 1:400. The immune responses of patients with pre-existing anti-NY-ESO-1 antibodies were judged as augmentation if the serum diluted 4-fold or more remained positive.

### Statistical analysis

Rates of the immune responses between the patients in Cohort 1 and Cohort 2 were compared by Fisher’s exact test, and the survival curve was estimated using the Kaplan–Meier method and compared by the log-rank test. In order to adjust the confounding factors, Cox proportional hazards model was applied. All analyses were done using SAS 9.2 (SAS Institute Inc., Cary, NC).

## Results and discussion

### Patient characteristics and clinical safety

A total of 25 patients were enrolled in the clinical trial. All patients had unresectable, advanced, or refractory esophageal cancers. The tumor cells in all of these patients were NY-ESO-1-positive, in which the positivity was determined by immunohistochemistry and qRT-PCR for 24 patients and one patient, respectively. All patients received standard chemotherapy and/or other cancer therapies including radiotherapy and surgery, which were ultimately ineffective (Table 1).

**Table 1:** Patients demographics

	**100 μg**	**200 μg**
No. patients enrolled	13	12
Sex		
Male	13	11
Female	0	1
Age		
Median	69	64.5
Range	49-72	53-79
Prior therapy		
Surgery	6	5
Radiotherapy	11	7
Chemotherapy	13	12
Pre-existing antibody to NY-ESO-1 antigen	3	7
No. vaccinations		
Median	8	9.5
Range	2-27	3-21

Cohort 1 consisted of 13 patients who were given 100 μg of the vaccine; Cohort 2 consisted of 12 patients who were given 200 μg of the vaccine. The patients in Cohort 1 and Cohort 2 received 2 to 27 vaccinations with a median of 8 doses and 3 to 21 vaccinations with a median of 9.5 doses, respectively (Table 1). No dose-limiting toxicity (DLT) was observed. All the patients except one developed transient, grade 1 skin reactions at the injection sites. Other adverse events included swallowing disturbance (n = 8), diarrhea (n = 3), and fever (n = 2), in which events of grade 3 or 4 were included. These events were considered unrelated to the CHP-NY-ESO-1 vaccination. Based on the laboratory data, decreased lymphocyte counts were observed (n = 10), which were all grade 3. These patients had lymphopenia at baseline, probably due to the previous chemotherapies. During the course of the vaccinations, they developed grade 3 lymphopenia, which were shifted from the other grade of the pre-vaccine lymphopenia. Other changes included decreased Na levels (n = 4), decreased hemoglobin levels (n = 3), elevated transaminase levels (n = 2) and elevated uric acid (n = 2) (Table 2). These adverse events were changed from the decreased or elevated levels at baseline. They did not affect the vaccine continuation. Therefore, the changes were considered not related or unlikely related to the vaccination.

**Table 2:** Adverse events during CHP-NY-ESO-1 vaccinations

	**100 μg(n = 13)**						**200 μg(n = 12)**						**Total**
**Adverse event**	**Grade**						**Grade**						
	**1**	**2**	**3**	**4**	**5**	**Subtotal**	**1**	**2**	**3**	**4**	**5**	**Subtotal**	
Skin reaction	12	0	0	0	0	12	12	0	0	0	0	12	24
Swallowing disturbance	0	0	3	0	0	3	0	0	4	1	0	5	8
Diarrhea	0	0	2	0	0	2	1	0	0	0	0	1	3
Fever	2	0	0	0	0	2	0	0	0	0	0	0	2
Decreased lymphocytes count	0	0	7	0	0	7	0	0	3	0	0	3	10
Decreased Na level	0	0	2	0	0	2	0	0	2	0	0	2	4
Decreased Hb level	0	0	3	0	0	3	0	0	0	0	0	0	3
Elevated ALT/AST level	0	0	2	0	0	2	0	0	0	0	0	0	2
Elevated uric acid level	0	0	1	1	0	2	0	0	0	0	0	0	2

NOTE: Events occurring more than once are listed. Events of disease progression are not listed.

### Immune responses to NY-ESO-1 protein

As shown Table 3, 3 out of the 13 patients, and 7 out of 12 patients had pre-existing antibodies to NY-ESO-1, while the remaining 10 and 5 patients did not have this reactivity in Cohort 1 and Cohort 2, respectively.

**Table 3:** Antibody responses in patients vaccinated with 100 μg or 200 μg of CHP-NY-ESO-1

**100 μg**				**200 μg**			
**pt No.**	**Vaccination cycle**	**Baseline (dilution titer)**	Antibody response (cycle*)	**pt No.**	**Vaccination cycle**	**Baseline (dilution titer)**	Antibody response (cycle*)
100-01	9	negative	responded(4)	200-01	15	negative	responded(2)
100-02	3	negative	no response**	200-02	9	negative	responded(2)
100-03	3	negative	no response**	200-03	8	positive (x1,600)	responded(5)
100-04	7	negative	no response	200-04	21	negative	responded(2)
100-05	2	negative	no response	200-05	3	negative	responded(2)
100-06	16	positive (x6,400)	responded(1)	200-06	10	positive (x400)	responded(1)
100-07	9	positive (x25,600)	no response	200-07	3	positive (x25,600)	responded(2)**
100-08	10	negative	responded(1)	200-08	11	positive (x400)	responded(1)
100-09	5	negative	no response	200-09	18	positive (x400)	responded(3)
100-10	27	positive (x400)	responded(3)	200-10	11	positive (x400)	responded(2)
100-11	8	negative	responded(2)	200-11	3	positive (x400)	responded(2)
100-12	8	negative	responded(2)	200-12	9	negative	responded(1)
100-13	26	negative	responded(2)				
antibody response rate		53.8%***			100%***		

*vaccine cycles with which antibody responses appeared. **antibody responses assayed after two vaccinations.***p = 0.015(Fisher’s exact test).

**Figure 1:**
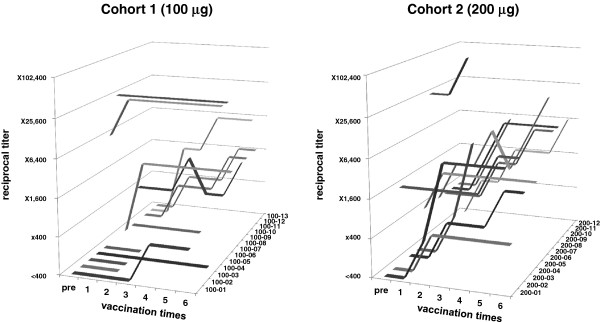
**Antibody responses to recombinant NY-ESO-1 protein as determined by ELISA.** Sera from 13 patients in Cohort 1 (100-μg dose) and 12 patients in Cohort 2 (200-μg dose) were collected at each vaccination, and serially diluted by 400, 1,600, 6,400, 25,600 and 102,400. Reciprocal titers were determined based on the maximally diluted sera, which showed a higher OD (optical density of 450–500) value than the cut-off level for NY-ESO-1 antibody positivity.

To evaluate the antibody responses after vaccination, serum samples collected at the serial vaccinations were analyzed using an antigen-specific IgG ELISA. In three patients of 100–02, 100–3 and 200–7 who were vaccinated three times, the serum samples from 1st and 2nd vaccination were assayed. In Cohort 1, out of 10 pre-antibody-negative patients, 5 became seropositive. Two out of 3 pre-antibody-positive patients had augmented antibody responses. In total, 7 of 13 (53.8%) patients exhibited immune responses. Five pre-antibody-negative and 7 pre-antibody-positive patients in Cohort 2 became positive or were augmented, yielding 12 out of 12 or 100% responsiveness. The 200-μg dose was more immunogenic than the 100-μg dose (p = 0.015, Fisher’s exact test). In Cohort 1, immune reactions were observed after a median of 2 cycles, with a range of 1 to 4 vaccine cycles. In Cohort 2, the immune responses were also evident after a median of 2 cycles with a range of 1 to 5 cycles (Table 3). The chronological appearance of the immune responses and antibody titers are shown in Figure 1. The antibody intensities appeared more quickly and at a higher titer in patients in Cohort 2 (200 μg) than those in Cohort 1(100 μg). In addition to His-tag NY-ESO-1 protein, we tested serum reactivities to GST-tag NY-ESO-1 protein and NY-ESO-1 peptides. We confirmed specific reactions to NY-ESO-1 antigen in these sera.

### Clinical responses and long-term follow-up

There were no cases of tumor shrinkage with partial response (PR) or complete response (CR) in any of the 25 patients. At the assessment that occurred every 6 weeks after vaccination, stable disease (SD) was observed in 3 patients in Cohort 1 and 6 patients in Cohort 2 (Table 4). There was no discordance in the evaluations between RECIST ver1.1 [11] and its modified version [12].

**Table 4:** Baseline clinical profiles and responses after CHP-NY-ESO-1 vaccinations

**100 μg**						**200 μg**					
**pt No.**	**Response to previous therapies (duration time, weeks)**	*sum of tumor diameters (mm)	**Tumor response (BOR)**	**Time-to-progression (weeks)**	**Survival (weeks)**	**pt No.**	**Response to previous therapies (duration time, weeks)**	*sum of tumor diameters (mm)	**Tumor response (BOR)**	**Time-to-progression (weeks)**	**Survival (weeks)**
100-01	PR (4)	NA	PD	6	31	200-01	PR (29)	24	SD	17	70
100-02	SD	53	NE	4	6	200-02	NE	25	SD	18	33
100-03	NE	144	NE	4	6	200-03	PR (32)	55	PD	6	37
100-04	PD	182	PD	5	17	200-04	PR (30)	NA	PD	6	50
100-05	CR (38)	101	NE	4	4	200-05	PR (32)	NA	PD	6	72
100-06	SD	69	PD	6	31	200-06	NE	32	SD	18	54
100-07	CR (15)	78	PD	6	29	200-07	NE	205	NE	6	8
100-08	NE	39	SD	18	23	200-08	PR (12)	16	SD	11	33
100-09	SD	18	PD	6	8	200-09	CR (96)	88	PD	6	45
100-10	CR (24)	NA	SD	11	60	200-10	SD	NA	SD	12	48
100-11	SD	31	SD	12	20	200-11	SD	NA	NE	6	12
100-12	PR (9)	NA	NE	16	28	200-12	SD	NA	SD	12	33
100-13	PR (16)	NA	NE	52	59						

*target lesions determined based on RECIST criteria.

**Figure 2:**
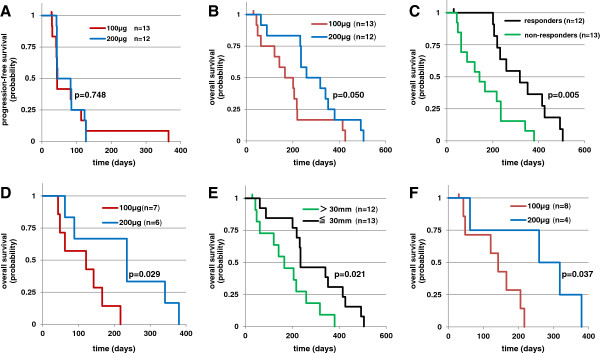
**Kaplan-Meier survival curves of patients who were alive after the CHP-NY-ESO-1 vaccinations. A**: Progression-free survival (PFS) of patients assigned to Cohort 1 (100-μg dose, n = 13) or Cohort 2 (200-μg dose, n = 12). The probability of PFS of the patients who received 200 μg of the vaccine and those who received the 100-μg dose were similar (log-rank test, p = 0.748). **B**: Overall survival (OS) of patients assigned to Cohort 1 or Cohort 2. The patients who received 200 μg of the vaccine survived longer than those who received the 100-μg dose. (log-rank test, p = 0.050). **C**: OS of patients vaccinated with either 100 μg or 200 μg of CHP-NY-ESO-1 compared between patients who responded to previous therapies and those who never responded. The patients who responded to previous therapies and were vaccinated with CHP-NY-ESO-1 survived longer than non-responders. (log-rank test, p = 0.0050). **D**: OS of patients vaccinated with 100 μg or 200 μg of CHP-NY-ESO-1 who never responded to previous therapies. The patients who received 200 μg of the vaccine survived longer than those who received the 100-μg dose (log-rank test, p = 0.029). **E**: OS of patients vaccinated with 100 μg or 200 μg of CHP-NY-ESO-1 compared with either baseline tumor burdens of >30 mm (sum of the tumor diameters) or 30 mm or less including non-measurable lesions. The patients with a tumor burden higher than 30 mm had reduced survival compared to those with smaller burdens (log-rank test, p = 0.021). **F**: OS of patients who received 100 μg or 200 μg of CHP-NY-ESO-1 who had baseline tumor burdens greater than 30 mm. The patients who received 200 μg of the vaccine survived longer than those who received the 100-μg dose (log-rank test, p = 0.037).

The disease progression-free survival time was 11 weeks on average, with a median of 6 weeks and range of 4 to 52 weeks. In Cohort 1 (n = 13), patients who were vaccinated with 100 μg of CHP-NY-ESO-1 survived without disease progression for 11 weeks on average, with a median of 6 weeks and range of 4 to 52 weeks. In Cohort 2 (n = 12) in which patients received the 200-μg dose, the patients were progression-free for 10 weeks on average, with a median of 8.5 weeks and range of 6 to 18 weeks (Table 4). There was no difference between the two cohorts (p = 0.748, Figure 2-A).

The overall survival time was 33 weeks on average, with a median of 31 weeks and range of 4 to 72 weeks. In Cohort 1 (n = 13), the patients survived for 25 weeks on average, with a median of 23 weeks and range of 4 to 60 weeks. In Cohort 2 (n = 12), they survived for 41 weeks on average, with a median of 41 weeks and range of 8 to 72 weeks (Table 4). The patients vaccinated with 200 μg of CHP-NY-ESO-1 had statistically longer survival than those who received the 100-μg dose (p = 0.050, Figure 2-B). Each cohort included three patients who were vaccinated three times or less because of early disease progression, and were withdrawn from this study, respectively. Having excluded those 6 patients, the patients vaccinated with 200 μg-vaccine still had longer survival than those with 100 μg-vaccinations (data not shown).

When the survival of patients who had responded to previous therapies (n = 12) was compared to non-responders (n = 13), the responders lived longer than the non-responders after vaccination (p = 0.005, Figure 2-C). The patients who never responded to previous therapies and received the 200-μg dose (n = 6) significantly lived longer than those who received the 100-μg dose (n = 7) (p = 0.029, Figure 2-D).

When the survival of patients who had tumors with a maximal diameter of 30 mm or less, including non-measurable lesions (n = 13) was compared with those with diameters more than 30 mm (n = 12), the patients with higher tumor burdens had shorter life spans (p = 0.021, Figure 2-E). Among patients with higher tumor burdens, patients who were vaccinated with the 200-μg dose (n = 4) lived longer than those who received the 100-μg dose (n = 8), (p = 0.037, Figure 2-F).

Using Cox proportional hazards models, the vaccine dose and the responsiveness to previous therapy were independent factors that influenced the overall survival, which showed p = 0.011 with HR 3.595 (95%CI 1.335-9.678) and p = 0.002 with HR 0.194 (95%CI 0.068-0.553), respectively. Also, the vaccine dose and the tumors sizes including non-measurable disease independently affected the overall survival, showing p = 0.040 with HR 2.630 (95%CI 1.045-6.614) and p = 0.020 with HR 0.322 (95%CI 0.124-0.833), respectively.

This study was a phase 1 dose-escalating clinical trial that examined two doses of the CHP-NY-ESO-1 vaccine in esophageal cancer patients. The primary goals were to evaluate the vaccine safety and immune responses to the NY-ESO-1 antigen, and we further explored the clinical effects on esophageal cancer patients with a poor prognosis.

CHP consists of a hydrophobic polysaccharide pullulan containing chemically introduced cholesterol groups, which spontaneously aggregate to form nano-sized particles that can contain antigen proteins. Using this system as a vaccine, tumor antigen proteins delivered to antigen-presenting cells can stimulate both antigen-specific CD4+ T cells and CD8+ T cells. In a pre-clinical study, dendritic cells pulsed with the CHP-NY-ESO-1 complex could induce both NY-ESO-1-specific CD4+ and CD8+ T cells [4]. Previous clinical studies using CHP-HER2 and CHP-NY-ESO-1 vaccines have shown that these vaccines can induce antigen-specific CD4^+^ and CD8^+^ T cell immunity in cancer patients [5-7].

In the current study, we found that CHP-NY-ESO-1 was clinically safe and that the immune responses to the NY-ESO-1 antigen, which were evaluated based on IgG antibody titers, showed a dose-dependent effect between the 100-μg dose and 200-μg. Furthermore, the survival rates of patients who were vaccinated with the 200-μg dose were superior to those who received the 100-μg dose. The patients had recurrent or metastatic esophageal tumors that exhibited clinical resistance to chemotherapy or radiotherapy. The first 13 patients were enrolled to Cohort 1, and the next 12 patients were included in Cohort 2. As the clinical backgrounds of the two cohorts were similar, it was reasonable to make a comparative consideration.

As the previous NY-ESO-1 protein vaccine trials have demonstrated, the toxicity of the CHP-vaccine was very mild. Grade 3 swallowing disturbances were seen, which were likely related to the progression of esophageal cancer. The other grade 3 events included diarrhea, which was not related to the vaccine. The only related events were grade 1 skin reactions at the injection sites.

Previous vaccine trials have used recombinant full-length NY-ESO-1 protein with various adjuvants. Melanoma patients were divided into three cohorts that were vaccinated with 10 μg, 30 μg or 100 μg of the NY-ESO-1 protein in combination with the saponin adjuvant ISCOMATRIX [10]. The 100-μg dose of NY-ESO-1 induced more immune responses than the other two doses. The responses were evaluated based on IgG antibody titers and delayed-type hypersensitivity (DTH) of skin reactions. In the CHP system, a single 100-μg dose of CHP-NY-ESO-1 was examined with or without the adjuvant OK-432 [6,7,16]. These reports suggested that the 100-μg dose of CHP-NY-ESO-1 is sufficient to induce immune responses. The current trial was designed to determine whether the NY-ESO-1 protein vaccine has potential dose-dependent effects on immunogenicity in patients with homogeneous backgrounds. By assessing humoral immune responses in the cohorts that received 100 μg and 200 μg of the vaccine, the responses appeared in the early phases. We initially intended to analyze antibodies using samples from patients who were vaccinated for at least 4 cycles, as we thought it could take at least 4 cycles to detect immune responses. In the overall data acquisition, samples from all 25 patients were analyzed, which included sera from at least two vaccinations. In conclusion, we found that the 200-μg dose was more efficient than the 100-μg dose.

The other reports included vaccine studies using recombinant NY-ESO-1 protein in combination with Imiquimod and CpG [17,18]. In these studies, the NY-ESO-1 protein was given at doses of 100 μg, and 100 μg or 400 μg, respectively. Based on the patients’ sera, the 400-μg dose might have induced more antibody responses than the 100-μg dose, but this was not statistically analyzed. Combined with these reports, the NY-ESO-1 protein might be immunogenic at increasing doses of 10 μg, 30 μg, 100 μg and 200 μg. Since dose-limited toxicity (DLT) was not observed at the higher dose of 200 μg in this study, additional dose increments might be acceptable to determine whether higher doses can induce stronger immune responses.

In this study, we explored a long-term clinical outcome of the NY-ESO-1 protein vaccine. This study was not initially designed to detect a statistical significance of the clinical effect between the 2 cohorts. Instead, we made a comparison to find out if there might include a positive signal for further clinical trials of this vaccine. The NY-ESO-1 protein vaccine with the adjuvant ISCOMATRIX suggested that melanoma patients who were vaccinated after standard therapy tended to have fewer relapses [10], which were not statistically analyzed. The other studies reported that vaccinations with NY-ESO-1-expressing poxvirus vectors and NY-ESO-1 overlapping peptides both prolonged progression-free survivals in ovarian cancer patients who did not have measurable disease after standard therapy [19,20]. In this study, most of the patients developed disease-progression in 6 months, and there was no difference between the patients vaccinated with 100 μg and 200 μg of the CHP-NY-ESO-1, as the previous studies demonstrated that disease-progression occurs in the early phase of vaccinations [12,21].

In contrast, we found that dose-dependent effects of the CHP-NY-ESO-1 vaccine on overall survival of patients with advanced/metastatic esophageal cancer. Analyzing other clinical categories, both the baseline tumor sizes and the tumor responsiveness to previous therapies were significant factors influencing the overall survival. Using Cox proportional hazards models, it was indicated that the tumor sizes and the vaccine doses independently influenced the survival. In the same way, the responsiveness to previous therapies and the vaccine doses independently affected the survival. Therefore, it is suggested that the higher dose of CHP-NY-ESO-1 vaccine played a role in prolongation of the overall survival in the esophageal cancer patients.

In addition, the higher-dose of the vaccine provided significant survival benefit in patients who never responded to the previous therapies or had larger tumor burdens than the lower dose vaccinations. It is difficult to discuss why the patients with a poorer prognosis were more benefited from the 200-μg dose of the vaccine than 100-μg. It might be speculated that the dose-dependency clinical benefits were more often observable in patients with a poorer prognosis, because they might have needed more immune responses in order to survive longer by preventing disease deterioration.

In the previous CHP-NY-ESO-1 vaccine study, which was a phase 1 study that enrolled various types of NY-ESO-1-expressing cancer patients, tumor regression was observed in two out of four esophageal cancer patients [6]. However, tumor shrinkage is rarely observed in cancer vaccine therapies, although some disease stabilization is seen. This study shows that clinical benefits, such as long-term survival, can be detected if a clinical trial is designed in a comparative way. The results were not compared to unvaccinated controls, and it is not possible to directly determine the effects of the vaccine, but is possible to reasonably interpret the effects of immune response on the clinical outcomes.

## Conclusions

The safety and immunogenicity of the CHP-NY-ESO-1 vaccine were confirmed in the patients with antigen-expressing esophageal cancer. The 200-μg dose efficiently induced antigen-specific immune responses and suggested better survival benefits, even for patients with a poorer prognosis. In future clinical trials, 200 μg will be the recommended dose.

## Abbreviations

BOR: Best overall response; NA: Not available; NE: Not evaluable.

## Competing interests

This study is supported by ImmunoFrontier, Inc. and Naozumi Harada is an employee, and Mami Ohnishi and Tadashi Hishida are former employees of ImmunoFrontier, Inc. Hiroshi Shiku is a stockholder of ImmunoFrontier, Inc.

## Authors’ contributions

SK, HW, KM, YN, SU, HM, ST and YD treated patients and provided the clinical data. SHS and YM worked on immune responses. HI, NI and ES evaluated tumor antigen expression. TY, MOs and MOh worked on the study statistics. NH and TH were responsible for manufacturing the study drug. SK and HS wrote the manuscript. All authors read and approved the final manuscript.
